# SARS-CoV-2 vaccination in Canadian blood donors: Insight into donor representativeness of the general population

**DOI:** 10.1016/j.jvacx.2024.100498

**Published:** 2024-05-12

**Authors:** Sheila F. O'Brien, Mindy Goldman, Behrouz Ehsani-Moghaddam, Wenli Fan, Lori Osmond, Chantale Pambrun, Steven J. Drews

**Affiliations:** a Epidemiology & Surveillance, Canadian Blood Services, 1800 Alta Vista Drive, Ottawa, Ontario K1G 4J5, Canada; b School of Epidemiology & Public Health, University of Ottawa, 600 Peter Morand Crescent, Ottawa, Ontario K1G 5Z3, Canada; c Donation and Policy Studies, Canadian Blood Services, 1800 Alta Vista Drive, Ottawa, Ontario K1G 4J5, Canada; d Department of Pathology & Laboratory Medicine, Faculty of Medicine, University of Ottawa, 600 Peter Morand Crescent, Ottawa, Ontario K1G 5Z3, Canada; e Centre for Studies in Primary Care, Department of Family Medicine, Queens University, 220 Bagot Street, Kingston, Ontario K7L 3G2, Canada; f Innovation & Portfolio Management, Medical Affairs & Innovation, Canadian Blood Services, 1800 Alta Vista Drive, Ottawa, Ontario K1G 4J5, Canada; g Microbiology, Canadian Blood Services, 8249-114 Street, Edmonton, Alberta T6G 2R8, Canada; h Department of Laboratory Medicine & Pathology, Faculty of Medicine & Dentistry, University of Alberta, 118 Street & 86 Avenue, Edmonton, Alberta T6G 2R3, Canada

**Keywords:** SARS-CoV-2, Blood donors, Vaccination, COVID-19

## Abstract

**Introduction:**

Blood donors world-wide were indispensable for monitoring anti-SARS-CoV-2 antibodies generated by infection and vaccination during the pandemic. Prior to the pandemic, donor vaccination behaviours were under-studied. We aimed to compare the percentage of Canadian blood donors with SARS-CoV-2 vaccination antibodies with the percentage of the general population who received at least one dose of vaccine each month during initial vaccine deployment. We also report donor attitudes towards SARS-CoV-2 vaccination.

**Methods:**

Canadian blood donors were randomly selected for SARS-CoV-2 antibody testing over 2021 (N = 165,240). The percentage of donor samples with vaccination antibodies were compared with the percentage of general population who received at least one dose of vaccine in each month of 2021 except February. A random sample of Canadian blood donors were surveyed about vaccination intent and attitudes (N = 4,558 participated, 30.4 % response rate).

**Results:**

The percentages of the general population vaccinated and donors with vaccination antibodies increased from 1 % to over 90 %. General population vaccination was greater early in vaccine deployment than donors (p < 0.05), greater in donors than the general population by mid-2021 (p < 0.05) but they were similar by the end of 2021. While 52.6 % of surveyed donors had received vaccine in May 2021, a further 41.1 % intended to when eligible. Most donors thought COVID-19 infection could be serious (83.5 %) and that it was important to be vaccinated even if previously infected (77.8 %).

**Conclusion:**

Early pandemic vaccine prioritization to at-risk individuals and healthcare workers gave rise to higher general population vaccination percentages, while donors had higher vaccine antibody percentages as vaccine was deployed to progressively younger age groups. Since blood donors may be more willing to receive vaccination, under pandemic conditions they may be valuable for monitoring vaccination-induced seroprevalence.

## Highlights

• Canadian adults were largely willing to receive COVID-19 vaccination when eligible.
• Blood donor vaccine-induced seroprevalence showed equal, if not better uptake of vaccination.
• Blood donors are valuable for monitoring vaccination-induced seroprevalence.

## Abbreviations

Anti-S: Antibodies to SARS-CoV-2 spike protein
Anti-N: Antibodies to SARS-CoV-2 nucleocapsid protein
Units/mL: Anti-S standard unit per milliliter
COI: Anti-N cutoff index

## Introduction

During the COVID-19 pandemic, countries world-wide turned to blood services to rapidly implement seroprevalence studies with the goal of informing public health policy [1]. This was successful and within the first year of the pandemic, blood donor vaccination uptake was integral to monitoring humoral immunity on a population level. Despite this success, a paucity of data on donor vaccination attitudes and behaviour in general was evident. Prior to the COVID-19 pandemic, there were some reports describing impacts of childhood vaccination on blood donor infection rates, notably hepatitis B [2], [3], [4], [5] and a few studies evaluating seroprevalence of childhood immunization in donors [6], [7], [8]. To our knowledge, there were no publications describing donor attitudes or behaviour regarding seasonal vaccination such as influenza.

In Canada all approved SARS-CoV-2 vaccines target the spike (S) protein [9]. Antibodies to spike protein (anti-S) are generated in response to vaccination, whereas antibodies to both nucleocapsid (N) and spike proteins form in response to natural infection. Lone anti-S can be considered a proxy for vaccination response, at least when infection prevalence is low. As part of the largest and longest running SARS-CoV-2 seroprevalence study in Canada [10], [11], [12], [13], our study monitored vaccine-generated anti-S in donors over the initial deployment of vaccine. Our study reported increasing positivity percentages in the oldest to progressively younger age groups which was consistent with public health policy targeting the oldest Canadians first [11]. Other countries have also monitored vaccine antibodies in blood donors during the pandemic [14], [15], [16], [17], [18], [19], [20] and some have commented on higher vaccine uptake in blood donors compared with general population vaccination at specific time points [21], [22]. A key gap is that other studies have not formally compared the uptake of COVID vaccine in blood donors vs the general population over time.

Here we compared the percentage of donors with SARS-CoV-2 spike protein antibodies with the percentage of the population who had at least one dose of SARS-CoV-2 vaccine over the first year of vaccine deployment in Canada. We also report results of a donor survey conducted mid-way through vaccine deployment about attitudes towards COVID-19 vaccination.

## Methods

### Canadian blood services donor base

Canadian Blood Services is responsible for collecting blood donations in all provinces in Canada except Quebec. Prior to donating blood, donors are required to answer screening questions to ensure they are in good health and are not at risk of transfusion transmissible infections. Donors are asked “In the last 3 months have you had a vaccination?” If yes, they are asked which infection they were vaccinated against. Currently, in Canada there is no deferral for receiving an influenza vaccine or SARS-CoV-2 vaccine. Donors also have their temperature checked to ensure they are afebrile before donating. To reduce risk of SARS-CoV-2 for donors and staff at the collection site, all donors were deferred from donating blood if they had been in contact with someone who was infected or if they had an infection for 2 weeks after symptoms disappear (3 weeks if hospitalized). For this study, donor age, sex, province and self-reported ethnicity data were extracted for 2021. Note that in this manuscript “vaccination uptake”is used as a general term to describe the proportion of people vaccinated.

### SARS-CoV-2 seroprevalence study

**Fig. 1:**
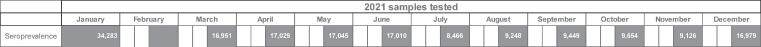
Number of samples tested per month, 2021.

The methods for SARS-CoV-2 seroprevalence are described elsewhere [11]. In brief, residual samples from donations were collected from approximately the last two weeks of every month from January 2021 to December 2021 as shown in Fig. 1. A straight random sample was applied up until June, after which samples were stratified into age groups by region before randomly selected while reducing the total monthly sample size.

All samples were tested using two assays: the Roche Elecsys® Anti-SARS-CoV-2 spike semi-quantitative immunoassay (Roche Diagnostics International Ltd, Rotkreuz, Switzerland) which measures total antibodies (including IgA, IgM, and IgG; units/ml [U/ml]) to the SARS-CoV-2 spike protein (anti-S) and the Roche Elecsys® Anti-SARS-CoV-2 qualitative immunoassay (Roche Diagnostics International Ltd, Rotkreuz, Switzerland) which measures total antibodies (including IgA, IgM, and IgG; cutoff index [COI]) to SARS-CoV-2 recombinant protein, nucleocapsid antigen (anti-N). At a concentration of ≥ 0.8 U/mL, the anti-S assay was assumed to have a sensitivity of 98.8 % and specificity of 99.97 % [23]. At a sample to cut off ratio of ≥ 1.0, the anti-N assay was assumed to have a sensitivity of 99.5 % and specificity of 99.8 % [24].

### General population vaccination history

The numbers of Canadians between the ages of 18 and 69 living in the nine provinces served by Canadian Blood Services were extracted from national statistical data tables [25]. The cumulative number of Canadians between the ages of 18 and 69 who had received at least one dose of COVID-19 vaccine each month of 2021 in the nine provinces served by Canadian Blood Services were extracted from national public health data tables [26].

### Survey

All whole blood donors negative for transmissible disease markers who had donated in the previous month and who had supplied an email address were eligible. Donors who had donated between March 27 to April 24, 2021 were invited by email to complete an on-line questionnaire between April 27, 2021 and May 11, 2021. Invited donors were sent a reminder email one week after the invitation email was sent. The questionnaire was developed by modifying a national telephone survey questionnaire, the Seasonal Influenza Vaccination Coverage Survey for an on-line method of administration focused on COVID-19 vaccination [27], [28]. The questionnaire included demographic questions, vaccination for COVID-19 or intention to be vaccinated when eligible, attitudes towards COVID-19 vaccination using a 5 point Likert scale and history of COVID-19 infection.

### Research ethics

This study was approved by the Canadian Blood Services Research Ethics Board (REB 2021.001). Informed consent was obtained for the survey. All donors in the seroprevalence study provided consent for donation which includes a battery of tests. Specific consent for SARS-CoV-2 testing was waived by the Canadian Blood Services Research Ethics Board.

### Statistical analysis

Data extraction, cleaning and analysis were carried out using SAS software (Cary, NC, USA, version 9.4 TS).

The cumulative percentages of general population who had received at least one dose of COVID-19 vaccine were calculated from general population cumulative vaccine frequencies and total population. Seroprevalence data were weighted by raking to reflect the age and sex distribution of the general population in the nine provinces served by Canadian Blood Services (Statistics Canada Data Catalogue number 98–400-X2016008), and adjusted for sensitivity and specificity of the assay using the Rogan Gladen equation [29]. The percentages of donors aged 18 to 69 who were anti-S positive and anti-N negative were assessed for each month. Data was displayed graphically with the percentage of general population who had received at least one dose of vaccine. For each month the percentages were compared using logistic regression. P-values less than 0.05 were considered to be significant.

As vaccine was available from late December 2020, COVID-19 vaccination could be assessed up until March 2021 with the vaccination in the last 3 months question. Donors in March 2021 who were anti-S positive were sorted into vaccinated and unvaccinated and the number and proportion anti-N reactive and anti-S reactive or lone anti-S reactive were calculated.

Frequencies and percentages were calculated for survey variables and for the full 2021 donor database where variables were available. Opinion questions asked on a Likert scale were simplified by combining agree and strongly agree into one response, and disagree and strongly disagree into another. To assess the impact of ethnicity on receiving vaccination or intent to be vaccinated, two logistic regression models were constructed: one with received vaccination and one with received vaccination or intend to when eligible as the dependent variable, and with age group, sex and ethnicity as the independent variables.

## Results

**Fig. 2:**
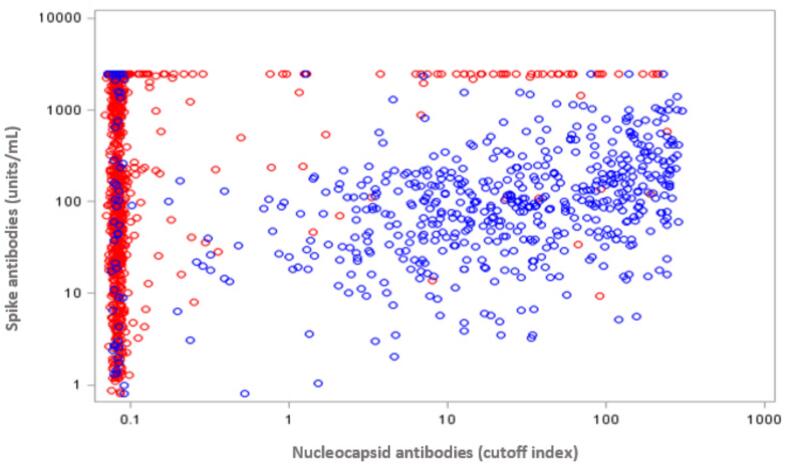
The concentration of vaccine(spike) antibodies in self-reported  vaccinated and unvaccinated blood donors in March 2021 . Note: As it takes up to 2 weeks to develop antibodies, donors who were recently vaccinated may have low or no anti-S.

**Fig. 3:**
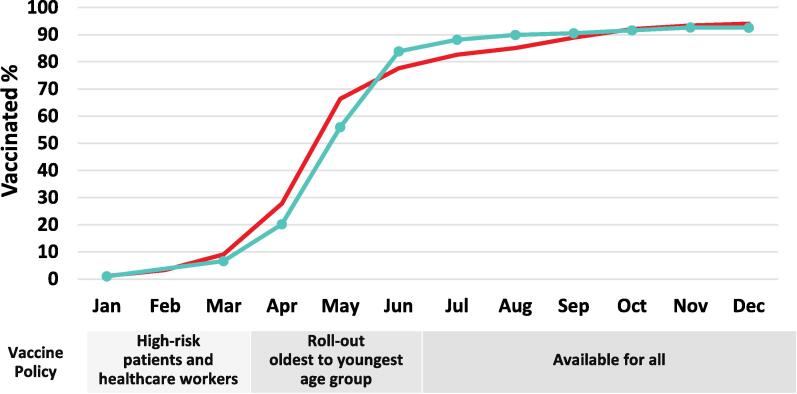
Percentage of general population aged 18–69 with at least one dose of vaccine  compared with the percentage of blood donors aged 18–69 with vaccine antibodies (Anti-S positive but anti-N negative) .

Between January 1, 2021 and December 31, 2021 a total of 165,240 blood donor samples were tested for SARS-CoV-2 antibodies. E-mail invitations were sent to 15,000 donors and completed by 4,582, for a response rate of 30.4 %. Fig. 2 shows a scatter plot of the antibody concentration among donors with anti-S (U/ml) positive results and their anti-N results (COI) in donations in March 2021, comparing those with self-reported vaccination and those where no vaccination history was reported. Most vaccinated donors (94.7 %) had anti-S but not anti-N, whereas most unvaccinated donors had both anti-N and anti-S (88.2 %). As shown in Fig. 3 the percentage of samples positive for anti-S was low in January (1 %) and increased between March and August to 89.9 % (92.6 % by December). The percentage of the general population with at least one dose of vaccine in the same age group increased similarly except there were more people in the general population with at least one dose of vaccine in January, March, April, May, November and December (p < 0.05) but more donors positive for spike antibodies in June, July, August and September (p < 0.05). The differences between donors and general population in January, November and December were very small (<0.2 %). Similar patterns were seen in each of the nine provinces included in the sample (Supplemental Fig. 1). The percentage of donors positive for antibodies to nucleocapsid is shown in Supplemental Table 1 where the percentage increased from 2.5 % in January, 4.2 % in May, 5.8 % in November and 7.1 % in December.

The donor demographic data and survey results are shown in Table 1. Survey participants were similar to all donors in 2021 for age group, sex and province but there were more White donors who participated in the survey. Most donors had post-secondary education, and of those with only high school 60 % were aged 19 or under. Nearly all donors (93.7 %) said they either had received at least one dose of the COVID-19 vaccine or were planning to once eligible. There were 6.4 % of donors who believed they had already had COVID-19, but of these only about a quarter had a positive test. Others based their assessment on symptoms. Nearly half of all donors in the survey (46.8 %) said they had been tested for COVID-19 at some point in the pandemic, and of these 42 % said it was because they had symptoms consistent with COVID-19 infection. Of the 6.4 % of donors who believed they had already had COVID-19, 87.4 % said they had been vaccinated or planned to get vaccinated when eligible. There were 28 % of donors who said someone they knew (family or friends) had tested positive for COVID-19 since the beginning of the pandemic.

**Table 1:** Responses to the survey questionnaire and demographics of the donor base in 2021.

	**Survey Participants**		**All Donors in 2021**	
	**n = 4,582**		**N = 360,533**	
	**n**	**%**	**n**	**%**
**Age Group**				
17–29	1,060	23.2	83,246	23.0
30–39	955	20.9	75,728	20.9
40–59	1,627	35.5	128,168	35.5
60+	937	20.5	74,390	20.6
**Gender**				
Male	2,198	48.0	174,211	48.3
Female	2,355	51.5	186,321	51.7
Non-binary	23	0.5		*
**Region**				
British Columbia	771	16.83	60,393	16.75
Alberta	842	18.38	66,196	18.36
Prairies	474	10.34	37,295	10.34
Ontario	2063	45.03	161,995	44.94
Atlantic	432	9.42	34,654	9.62
**Education level**				
High school or less	448	9.9		
Trade/vocation (includes community college)	1,079	23.7		
University (some or completed)	2,097	46.1		
Post grad	922	20.3		
**Number of people in household**				
One	929	20.3		
Two to five	3,518	76.9		
Six or more	125	2.7		
Ethnicity **				
White	3,857	84.3	88,686	72.4***
Indigenous	109	2.4	2,113	1.7
Asian	384	8.4	18,880	15.4
Other Racialized Group	226	4.9	12,840	10.5
**Country of Birth**				
Canada	3,898	85.1		
Other	683	14.9		
**Has had Covid-19**	288	6.4		
**Have you had Covid-19 vaccine**				
Yes	2,375	52.6		
No, but planning to	1,853	41.1		
*Donors in the donor base do not have the option of selecting non-binary **Total number of CBS donors is lower for ethnicity question than for other demographics because it is optional for donors to answer. ***p < 0.05				

**Fig. 4:**
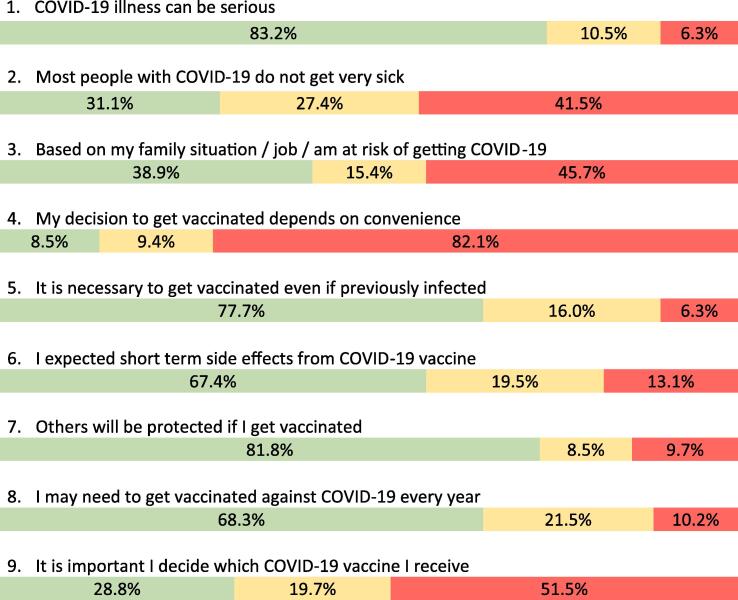
Donor attitudes towards COVID-19 vaccination in May 2021.  Agreed  Undecided  Disagree.

Fig. 4 shows the responses to attitude/opinion questions. Most donors believed that COVID-19 could be a serious disease but less than half thought that most people got very sick. About half thought they had considerable risk of getting COVID-19. Most donors thought it was important to get vaccinated even if previously infected, only 6 % said it was not important. Side effects were expected by most, most believed they would need to be vaccinated every year, and most believed that if they were vaccinated it would also protect others. Ethnicity was not associated with having been vaccinated (p = 0.89) or having been vaccinated/intend to be vaccinated when eligible (p = 0.82).

## Discussion

Prior to the COVID-19 pandemic, vaccination in donors was under-studied. Blood-banking interest was largely limited to deferral of donors with recent live-attenuated vaccination such as for measles for blood recipient safety. There were donor selection criteria to avoid false positive infectious markers such as from recent hepatitis B and Japanese encephalitis virus vaccines [30]. There were some reports on infection prevalence in blood donors stratified by age eligibility for receiving childhood vaccinations. These were primarily relating to chronic hepatitis B infections, mostly oriented towards evaluating blood safety [3], [4], [5] although we recently evaluated the value of such data for public health policy [2]. There are a few examples of blood donor studies to inform public health policy estimating seroprevalence of childhood vaccination in adults. For example, a 2012 study in one hospital blood bank in Italy measured antibodies to measles [6], a 2015 study in Rwanda reported donor data on rubella antibodies [8], and a donor subgroup was included in a 2013–2015 sero-survey of whooping cough antibodies in Laos [7]. For the most part blood service participation in public health surveillance was limited to reporting operational data such as positive tests from routine blood screening.

Shortly after the WHO declared a pandemic in March 2020 [31], blood services around the world took the unprecedented step of launching sero-surveys to inform public health policy [1]. With the deployment of vaccines less than a year later, blood donor vaccination uptake was integral to monitoring humoral immunity. In Canada the first COVID-19 vaccine was authorized on December 9, 2020 and the first vaccine was administered December 14, 2020 [32]. Vaccine was initially prioritized to long-term care residents and high-risk patients, followed by healthcare workers. By March 2021 most provinces began rolling out vaccine to their oldest residents, and then progressively younger age groups. Vaccines were available from several manufacturers, but people often had limited or no choice which they received.

Our study is the largest and longest running SARS-CoV-2 seroprevalence study in Canada [10], [11], [12], [13]. The humoral response to vaccination was monitored in blood donors in many countries including the United States, Germany, the Netherlands, Wales, Ireland, Austria and South Africa [14], [15], [16], [17], [18], [19], [20]. Blood donor cohort studies have provided valuable insight into antibody waning and hybrid immunity [33], [34]. The global reliance on blood services to inform public health policy through donor sero-surveillance heralds a new role for blood services post-pandemic [35].

Understanding health behaviours in blood donors relative to the general population is essential to interpreting donor data for public health purposes. Donors have been reported to have healthier lifestyles such as exercising more, smoking less and better perceived health than non-donors in Denmark and the Netherlands [36], [37], [38]. The “healthy donor effect” is believed to be a combination of self-selection to donate and donor health criteria [39]. However, donor criteria and population perceptions of what it means to be healthy vary by country. Most European countries have an upper age limit and defer for health conditions such as insulin-treated diabetes, history of coronary artery disease or cancer, whereas other countries such as Canada and the United States do have no upper age limit and have less stringent health criteria. Therefore, each country needs to understand donor health and health-related behaviours in their own country.

General population uptake of vaccine by the end of 2021 varied by country with only 73 % in the US [40], in Germany and the Netherlands 85 %, and in France 93 % [41]. Canada was among the highest [40]. The stronger the general population uptake of vaccination, the less latitude there is to observe a difference in donors, although higher vaccination rates in donors were observed at specific time points in the US and France [21], [22], [42].

In our study an important distinction must be made between donor and general population data. In donors antibody response to vaccination was measured, but in the general population vaccination receipt was measured. With SARS-CoV-2 infection, antibodies develop to both spike protein and nucleocapsid, whereas after vaccination only antibodies to spike protein will form. We report a correlation between vaccination and anti-S positive/anti-N negative results early in vaccine roll-out, although important limitations were self-reported vaccination and we did not ask about COVID-19 infection history. We analyzed the percentages of donors who had anti-S without anti-N, but it is possible that some donors with anti-S had been infected but did not develop anti-N antibodies or anti-N waned to undetectable levels [43]. As vaccination antibodies can take up to two weeks to develop, it is also possible that some recently vaccinated donors were not detected by the anti-S assay [44]. Most Canadians and most donors in our survey believed it was important to be vaccinated against COVID-19 even if previously infected [45], [46]. We considered the possibility that we underestimated the percentage of donors with vaccine antibodies by not counting those with evidence of past infection. In January 2021 2.5 % of donors had anti-N from infections prior to deployment of vaccine. In May there were 4.2 %. Even if all 4.2 % were in the half of donors eligible for vaccine at that time and had all been vaccinated, it would not account for the 10.4 % difference between donors and general population in May. Similarly, the percentage anti-N positive increased a further 0.3 % in August, but donors were then 4.8 % higher than the adult general population. By the end of 2021 7.1 % of donors had been infected of whom most would have been vaccinated, thus likely the true percentage of vaccinated donors was slightly higher than the general population.

The percentage of Canadian blood donors with COVID-19 vaccine (anti-S) antibodies increased dramatically in 2021 concomitant with vaccine deployment. Early in vaccine deployment (up to May) the percentage of donors with vaccine (anti-S) antibodies was somewhat less than the percentage vaccinated in the adult general population. Vaccination was deployed to older, then progressively younger age groups, but also prioritized to individuals at high risk of complications such as immunocompromised individuals and health care workers. Vaccine was also prioritized to long term care residents but most would not be included because in our analysis the age group was limited to 18–69 years. Due to underlying health concerns high risk individuals may be ineligible (such as those undergoing treatment for cancer or with primary immune deficiency) or less likely to donate (such as immunocompromised individuals advised to avoid risk of SARS-CoV-2). The high pandemic demand for health care workers may have limited their ability to attend a donor clinic. While only about half of donors in our survey (in May) had received the vaccine, 94 % expected to be vaccinated once eligible. This was slightly higher than 89 % reported in a general population survey in April/May 2021 [45], [46], [47]. Donor vaccination was higher than the general population by June when all adults were eligible. Taken together, while donors were less likely to be vaccinated during early vaccine deployment due to public health prioritization policies, they were keener to be vaccinated when eligible. By the fall of 2021 most jurisdictions in Canada had a vaccine passport system in which entry to most public venues including restaurants, gymnasiums, schools and many workplaces required proof of vaccination. This likely encouraged vaccination over that period, plus vaccine was more readily available [48].

It is important to note that while we were able to adjust the blood donor seroprevalence results to be similar to the general population for age, sex and region, there may be other differences that could not be adjusted for such as ethnicity, occupation socioeconomic status for which donors may not be fully representative [49]. As differences between donor anti-S seroprevalence and general population were small we cannot rule out the possibility that differences could be due to extraneous variables or to the use of two different kinds of data (general population vaccination history vs donor anti-S seroprevalence). We were unable to distinguish between donors who received one or two doses of vaccine, hence limited the analysis to comparison with general population who had at least one dose.

Overall donors expressed good knowledge and positive attitudes towards the COVID-19 vaccine. There was understanding that COVID-19 could be serious even though many people may experience mild illness, and that vaccination was necessary even if previously infected. Even though only about half thought they were at risk of getting COVID-19, nearly all were or intended to be vaccinated. This is similar to 45 % of adults vaccinated and 49 % intending to be vaccinated in a general population study carried out at the same time as ours [48]. Importantly, there was strong agreement that through vaccination they were protecting themselves and others similar to a general population survey [45], [46], [47]. This sentiment of protecting self and others was encouraged by public health messaging at the time, and appears to be reflected in strong uptake of vaccine among donors and the general population. In Canada messaging was communicated to the public through frequent newscast featuring senior public health physicians and through websites and through community groups. There may be a range of factors that affect vaccine hesitancy that vary across regions [50]. Among blood donors high vaccine uptake likely provided important benefits to the sufficiency of the blood supply since vaccinated donors would be less likely to be sick and unable to donate, plus vaccinated blood donors posed less risk to other donors and staff in the blood collection site.

In summary, we report modest variation in COVID-19 vaccination behaviour between blood donors and the general population. The overall strong acceptance of vaccination by Canadians was reflected in donors, who appear to be amongst the most willing to step up for vaccination. Our results suggest that under pandemic conditions Canadian blood donors were reasonably representative of Canadian vaccination uptake, and donor seroprevalence data will continue to be valuable for monitoring the humoral response to both vaccination and infection.

**Availability of Materials**

Data in summary form and the questionnaire can be made available by contacting Sheila O’Brien (sheila.obrien@blood.ca).

**Funding Source**

This study was funded by the Government of Canada through the COVID-19 Immunity Task Force. The funding source had no involvement with the study design, collection, analysis or interpretation of data.

## CRediT authorship contribution statement

**Sheila F. O'Brien:** Conceptualization, Formal analysis, Methodology, Writing – original draft. **Mindy Goldman:** Formal analysis, Writing – review & editing. **Behrouz Ehsani-Moghaddam:** Data curation, Formal analysis, Writing – review & editing. **Wenli Fan:** Data curation, Formal analysis, Writing – review & editing. **Lori Osmond:** Data curation, Formal analysis, Visualization, Writing – review & editing. **Chantale Pambrun:** Conceptualization, Funding acquisition, Writing – review & editing. **Steven J. Drews:** Conceptualization, Formal analysis, Writing – review & editing.

## Declaration of competing interest

The authors declare the following financial interests/personal relationships which may be considered as potential competing interests: Chantale Pambrun reports financial support was provided by Government of Canada. Steven Drews reports equipment, drugs, or supplies was provided by Abbott Laboratories. Steven Drews reports a relationship with F Hoffmann-La Roche Ltd that includes.

## Data Availability

The data that has been used is confidential.
