# Supplementary material for: SARS-CoV-2 vaccination in Canadian blood donors: Insight into donor representativeness of the general population
Source: Vaccine X. 2024 May 12;18:100498. doi: 10.1016/j.jvacx.2024.100498 (PMC11127215; doi:10.1016/j.jvacx.2024.100498)
Supplement: Supplementary Data 1 [file mmc1.docx]

**Supplementary Table 1 Anti-nucleocapsid seroprevalence in blood donors in 2021**

| **Month** | **Number tested** | **Percentage Positive** | **95% CI** |
| --- | --- | --- | --- |
| January | 34,283 | 2.47 | (2.31-2.63) |
| February***** |  |  |  |
| March | 16,951 | 3.44 | (3.16-3.71) |
| April | 17,029 | 3.29 | (3.03-3.56) |
| May | 17,045 | 4.17 | (3.87-4.47) |
| June | 17,010 | 4.77 | (4.45-5.09) |
| July | 8,466 | 4.43 | (3.99-4.87) |
| August | 9,248 | 4.55 | (4.13-4.98) |
| September | 9,449 | 4.71 | (4.28-5.14) |
| October | 9,654 | 4.79 | (4.36-5.21) |
| November | 9,126 | 5.79 | (5.31-6.26) |
| December | 16,979 | 7.08 | (6.69-7.47) |
| *****samples were not tested in February | | | |
