# Supplementary material for: SARS-CoV-2 vaccination in Canadian blood donors: Insight into donor representativeness of the general population
Source: Vaccine X. 2024 May 12;18:100498. doi: 10.1016/j.jvacx.2024.100498 (PMC11127215; doi:10.1016/j.jvacx.2024.100498)
Supplement: Supplementary Data 2 [file mmc2.docx]

**Supplemental Figure 1** Percentage of general population aged 18-69 with at least one dose of vaccine
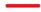
compared with the percentage of blood donors aged 18-69 with vaccine antibodies (Anti-S positive but not anti-N positive)
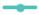
 in nine provinces where blood is collected.


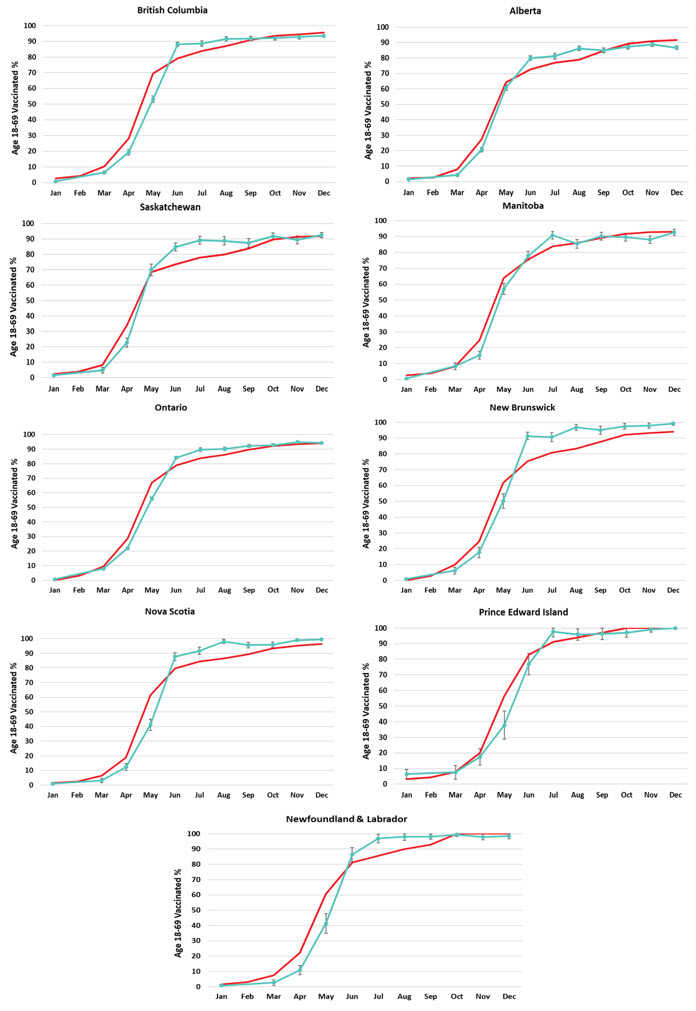


**
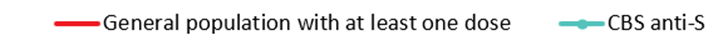
**
